# Cognitive versatility and adaptation to fluid participation in hospital emergency department teams

**DOI:** 10.3389/fpsyg.2024.1144638

**Published:** 2024-02-27

**Authors:** Ishani Aggarwal, Anna T. Mayo, Toshio Murase, Evelyn Y. Zhang, Brandy Aven, Anita Williams Woolley

**Affiliations:** ^1^Brazilian School of Public and Business Administration, FGV, Rio de Janeiro, Brazil; ^2^Heinz College of Information Systems and Public Policy, Carnegie Mellon University, Pittsburgh, PA, United States; ^3^School of Commerce, Waseda University, Tokyo, Japan; ^4^Nanyang Business School, Nanyang Technological University, Singapore, Singapore; ^5^Tepper School of Business, Carnegie Mellon University, Pittsburgh, PA, United States

**Keywords:** Carnegie School, fluid participation, teams, cognitive style, roles, team composition

## Abstract

Role-based frameworks have long been the cornerstone of organizational coordination, providing clarity in role expectations among team members. However, the rise of “fluid participation”—a constant shift in team composition and skill sets—poses new challenges to traditional coordination mechanisms. In particular, with fluid participation, a team’s roles can oscillate between disconnected and intersecting, or between lacking and having overlap in the capabilities and expectations of different roles. This study investigates the possibility that a disconnected set of roles creates a structural constraint on the flexible coordination needed to perform in volatile contexts, as well as the mitigating role of cognitive versatility in a team’s strategically-central member. Utilizing a sample of 342 teams from a hospital Emergency Department, we find that teams with a disconnected role set are less effective than teams with an intersecting role set as demonstrated by longer patient stays and increased handoffs during shift changes. Importantly, the presence of a cognitively versatile attending physician mitigates these negative outcomes, enhancing overall team effectiveness. Our findings remain robust even after accounting for other variables like team expertise and familiarity. This research extends the Carnegie School’s seminal work on fluid participation by integrating insights from psychology and organizational behavior, thereby identifying key individual attributes that can bolster team coordination in dynamic settings.

## Introduction

As management scholars have established over the last several decades, any form of organizing must solve two fundamental and interlinked problems—the division of labor and the integration of effort (March and Simon, 1958; Puranam et al., 2014). The foundational work of the Carnegie School, including their major pillars of bounded rationality, routine-based behavior, and learning, has resulted in the adoption of many essential mechanisms for addressing these basic problems of organizing. Examples include the use of role-based structures (e.g., Bechky, 2006) and protocols or standard operating procedures (e.g., Faraj and Xiao, 2006), which have served as important anchors for enabling the effective division and integration of labor in many organizational settings, particularly when groups need to assemble and respond to a range of planned and unplanned events.

However, it has become more challenging for traditional structures and routines to facilitate effective organizing given the increasing complexity of the environments organizations work within (Edmondson and Harvey, 2018). Many organizations began to adopt team-based forms of organizing in the 1980s in order to facilitate the adaptation needed to solve problems and carry out work, but scholars have noted a sharp increase in the fluidity of even these team structures, where the boundaries of a work unit are increasingly hard to identify and the problems of organizing more and more difficult to solve across an ever-changing cast of contributors (Humphrey and Aime, 2014; Mortensen and Haas, 2018; Mayo, 2022). The result can bear a strong resemblance to the organized anarchies articulated by Cohen et al. (1972), which are characterized, in part, by what they termed “fluid participation.” Even in these more dynamic environments, research following Cohen et al.’s (1972) work has explored how organizational structures can support individuals’ ability to adapt to the conditions of organized anarchy (Cohen et al., 2012). In adjacent literature, research on fluid participation in teams has similarly demonstrated the value of structural elements such as “structured role systems” (Bechky, 2006) or “de-identified role sets” (Valentine and Edmondson, 2015), whereby clear expectations for a defined set of roles (e.g., a nurse and physician in a healthcare setting) enable coordination despite fluid participation. However, the ever-increasing dynamism characterizing many work contexts renders even the practice of structured role sets inadequate, as fluid participation often comes with a changing skill set configuration across members (Bechky and Okhuysen, 2011; Mayo, 2022), and this could alter the extent to which roles overlap in the tasks that they could do, yielding what we refer to as a more-or less-connected role set.

Here we suggest that in light of the weakening of structural elements, such as roles and routines, traditionally relied upon to organize work, team members’ individual characteristics are likely to become an increasingly important influence on a team’s ability to coordinate effectively in the face of fluid participation. Specifically, we build on recent research to theorize that teams needing to adapt to fluid participation can benefit from team members’ cognitive versatility, a quality characterizing individuals who have flexibility in thinking style for acquiring, organizing, and processing information (Ausburn and Ausburn, 1978; Aggarwal et al., 2019, 2023). The extant literature on cognitive styles has demonstrated the benefits of the ability to shift between cognitive styles for individual flexibility and adaptation to change (Kozhevnikov et al., 2014). Recent research also explored the benefits of individual cognitive versatility in the context of teamwork, finding that the presence of cognitively versatile members facilitates the task and social processes necessary for effective team information processing, leading to better performance (Aggarwal et al., 2023). We consider these observations alongside related work on team composition demonstrating the outsize influence of central or “core” team members (Humphrey et al., 2009), such that their characteristics are particularly influential for team outcomes (Mathieu et al., 2014; Emich et al., 2022). We integrate these arguments to theorize that the cognitive versatility of core members can enhance a team’s ability to coordinate in the face of fluid participation, particularly under conditions that require a team to operate with a less-connected role set.

We test our theory related to the benefit of cognitively versatile members in a sample of 342 teams working in an Emergency Department (ED) in a medium-sized suburban hospital in the U.S. The *more-connected role set* for staffing teams on each shift included an attending physician, a nurse practitioner, and an average of seven nurses. However, in approximately half of the teams, there was not a nurse practitioner included, forcing those teams to operate with a *less-connected role set*. Even if the same number of team members were involved, the inclusion of the nurse practitioner role offered teams additional flexibility as members with that role can perform nursing duties as well as most of the duties of the attending physician (while working on a team supervised by an attending physician). Therefore, operating with a less-connected role set reduced the level of flexibility a team could exercise in their coordination. The results confirmed our predictions that less-connected role sets are associated with less team effectiveness as indexed by the efficiency of care teams provided, reflected in longer length of stay in the ED and the number of patients handed off to the next team during a staffing shift change. However, if a team’s attending physician—considered to be the strategically-core member—was more cognitively versatile, the team provided more efficient care overall, and was less negatively affected by working with a less-connected role set compared to teams with less cognitively versatile attending physicians. Effects remained robust after accounting for other potential explanations, such as team-member familiarity and the attending physician’s prior job experience, along with other team member characteristics shown to be beneficial to teamwork in prior studies. This work contributes to the Carnegie School tradition by identifying attributes of team members which can complement team structure to enable effective coordination.

## Theoretical background

### The Carnegie School, attention and fluid participation

Among the variety of foundational concepts emerging from the Carnegie School is “the notion that the organization of attention is a central process out of which decisions arise” (DiMaggio and Powell, 1991, p. 19). Indeed, early work within this tradition by Simon and March challenged dominant views of a rational choice model by introducing the concept of bounded rationality, which emphasized attentional limits that constrain our understanding of problems and solutions (March and Simon, 1958; Simon, 1997). As Simon writes, “rationality requires a choice among all possible alternative behaviors. In actual behavior, only a very few of all these possible alternatives ever come to mind” (Simon, 1997, p. 81). This attention to attention has had a wide-reaching influence that spans disciplines, impacting, for example, the study of cognitive biases and heuristics in individual decision making (e.g., Tversky and Kahneman, 1981; Bazerman and Moore, 2012) as well as the development of an attention-based view of the firm (Ocasio, 1997). Moreover, these ideas are foundational to the argument that organizational structures can guide our limited attention and thereby support coordination (March and Simon, 1958).

Like many others who picked up on the importance of attention, the concept was integrated into Cohen et al.’s (1972, p. 2) “garbage can model of organizational choice,” in which the authors note the need to “understand the attention patterns within organizations.” Influenced by writings such as Lindblom’s essay on “muddling through” (Lindblom, 1959), Cohen and colleagues departed from some of the Carnegie School’s traditional assumptions of the rational decision-making model to introduce the notion of an “organized anarchy,” characterized by goal ambiguity, solutions searching for problems, and fluid participation. In developing their theory, their fundamental insight was to disentangle solutions from problems and propose that—far from rational—decision-making is the result of the temporal coupling of participants, solutions, problems, and choice opportunities. Notably, fluid participation’s contribution to organized anarchy is via the ways it limits the attention that participants can direct toward decisions (Ocasio, 2012) even in *stable* membership environments. Elements of the garbage can model have had a lasting impact on the subsequent study of decision-making, spanning disciplines from education to political science, public administration, management, and sociology (Jann, 2015), and it is still the focus of special issues of journals even 40 years after its introduction (Lomi and Harrison, 2012). However, most of the work building on the garbage can model has centered on mitigating or adapting to goal ambiguity (Cohen et al., 2012; Ocasio, 2012) while the role of fluid participation remains relatively under-developed (c.f., Ganz, 2021).

We argue that the concept of fluid participation has become increasingly important to understanding the challenges many organizations face, and particularly as it relates to teamwork. Organizational use of team structures accelerated starting in the 1980s as technological advances allowed for more rapid sharing of information that could support decentralized decision-making and thus more agile responses to complex and volatile environments (Malone, 2004). And as team structures become increasingly fluid, we see parallels between work demonstrating the support of intelligent action within an organized anarchy via structures to guide attention, as described in the garbage can model (e.g., see review in Cohen et al., 2012), and the benefits of mechanisms like role structures to guide attention in temporary teams with fluid participation (Bechky, 2006; Valentine and Edmondson, 2015). In drawing these parallels, we see an opportunity to further develop the original conception of fluid participation from the garbage can model and connect it with extant work on temporary, role-based teams that experience increasing fluidity in participation.

Fluid teams are characterized by changing sets of participants working on a shared task (Hackman and Wageman, 2004; Humphrey and Aime, 2014; Mortensen and Haas, 2018), where the number and configuration of skill sets vary over time (Mayo, 2022). Teams experience increasing fluidity in response to a variety of conditions, including labor shortages, conflicting priorities, double-booked schedules, or (un) scheduled absences from work. Even when a team has the usual number of members, sometimes the configuration of skills across members can vary, requiring team members to adapt their role structure. Thus, even when the required number of people and expertise is available, the coordination patterns need to change if, for example, a team now has one team member handling some tasks that used to be done by two different team members, or if different tasks that were done together by one member now need to be separated and handled by different members. Given this state of work in many organizations, in the parlance of the garbage can model, decisions might arise from the confluence of fluid participation and the loosely coupled choice opportunities (e.g., the need to allocate attention given who is currently available), problems (e.g., the need to reconfigure coordination due to changing role sets) and the available solutions (e.g., as identified based on the cognition of whoever happens to be involved in the work at that given time). In building on extant work to seek solutions to facilitate adaptation to the current state of “anarchy” and thus support more intelligent action (e.g., see review in Cohen et al., 2012) in fluid teams, we draw on research in adjacent literatures including psychology and organizational behavior to further identify ways teams can adapt to changing role structures. In doing so, we draw on work on the features of team design that can guide attention, specifically team composition.

### Team composition: cognitive versatility in the strategic core

A team’s composition, or the mix of its members, is one of the key levers available for impacting the team’s processes and thereby team effectiveness (Bell et al., 2018). Studies in this area generally consider how the combination of team members’ attributes (including demographic characteristics as well as other personal traits) influence team effectiveness (e.g., Loyd et al., 2013; Riedl et al., 2021; Emich et al., 2022). Research on team composition has developed over several decades and has evolved from considering strictly task-relevant abilities to considering other traits and characteristics that affect team collaboration (Mathieu et al., 2014). Extant work has examined a variety of individual characteristics considered to be relatively stable traits that influence behavior across situations, such as personality, or cultural values, and more recently attention has turned to cognitive style. Cognitive styles capture stable tendencies in how individuals “acquire, organize and process information” (Ausburn and Ausburn, 1978; Aggarwal et al., 2019). Cognitive styles can drive how people learn and the approaches they take to problem-solving, including the solutions they conceptualize (e.g., see Kozhevnikov et al., 2014) and the ways they coordinate with team members (Aggarwal and Woolley, 2013), making them a significant influence in many areas of work. One framework developed initially by cognitive neuroscientists identifies three distinct cognitive styles (object visualization, spatial visualization, and verbalization) that affect an individual’s facility with, and preference for, distinct ways of encoding, presenting, and processing information (Kozhevnikov et al., 2002). As summarized by Kozhevnikov et al. (2014), individuals who are strong in object visualization think more holistically, processing and communicating information by using detailed pictorial images of objects. In contrast, those who are strong in spatial visualization are more analytical than holistic in their thinking, processing and communicating information with images, but with an emphasis on the spatial relations among parts of the whole. Lastly, those strong in verbalization are also more-analytical thinkers and also break information down into parts and their relations, but they tend to encode, process, and express it verbally rather than in images, facilitating processes such as analogical reasoning. Cognitive styles have been shown to emerge in early childhood based on innate tendencies, which are reinforced by associated choices of hobbies, school coursework, and occupation (Blajenkova et al., 2006; Kozhevnikov et al., 2010).

Initial research on cognitive styles in teams explored the impact of having members with diverse cognitive styles, finding that a mix of cognitive styles was essential for problem-solving (Woolley et al., 2007) and that cognitive style diversity among members enhanced teamwork by providing cues to complementary strengths and facilitating the development of transactive memory systems (Aggarwal et al., 2019). It is also the case that team cognitive style diversity can create coordination difficulties by reducing strategic consensus (Aggarwal and Woolley, 2013) and, if not managed well, maintains a non-monotonic, inverted-U shaped relationship with collective intelligence such that the benefits of a moderate level of cognitive style diversity are reversed at the highest levels (Aggarwal et al., 2019).

Furthermore, where possible, coaching interventions to help teams make use of their complementary strengths can facilitate high performance, even in highly cognitively diverse teams (Woolley et al., 2008). However, coaching a team effectively requires more stability in team membership than is the reality in a growing number of work contexts (Mayo, 2022). Consequently, some have turned to considering other qualities of team members themselves that might help teams benefit from the diverse skills of members. One related area of research has considered whether within-team diversity might be facilitated by within-person cognitive diversity, building on studies demonstrating that some individuals exhibit flexibility in cognitive style, also termed cognitive versatility (Aggarwal et al., 2023). As observed by Kozhevnikov et al. (2014), an individual’s diversity and flexibility in cognitive styles can lead them to be able to adapt to and learn in different situations. Across a variety of studies examining within-person or intrapersonal diversity, research demonstrates that individuals who are more intrapersonally diverse facilitate collaboration and performance within diverse teams (e.g., Bunderson and Sutcliffe, 2002; Mok and Morris, 2010; Lu et al., 2022) even when they do not share specific diversity traits in common with other members (Jang, 2017). This seems to occur, in part, because they themselves are more flexible and creative, as extant research has demonstrated that cognitive versatility is associated with creativity (Meneely and Portillo, 2005; c.f., Ho and Kozhevnikov, 2023) as well as efficiency in problem solving due to an ability to adapt one’s strategies to a given situation (for review, see Kozhevnikov, 2007). In addition, recent research demonstrates that the presence of cognitively versatile individuals enhances social integration in teams, leading teams to experience less task and process conflict, and better team performance (Aggarwal et al., 2023). Taken together, the evidence suggests that teams benefit from the presence of cognitively-versatile members who are more flexible and creative in solving problems as well as able to convey ideas and plans in ways that facilitate the comprehension and cooperation of diverse team members. However, since most individuals operate predominantly with one cognitive style (Kozhevnikov et al., 2014), it is unlikely that organizations can create teams with members who are all cognitively-versatile. Thus an important question is how many of these members are needed, or is there a way to use these unusual contributors to best effect?

We consider the question of how best to use cognitively-versatile members by integrating literature on team composition with related work that examines team structure using a network lens. Specifically, research over the last decade or so suggests that teams working in dynamic environments often organize into patterns around one or a small group of members who, from a networks lens, are central to the work and information flow, typically involving decision-making authority (Ancona and Bresman, 2007; Humphrey et al., 2009). Such members are considered strategically “core” members, and often coordinate with a variety of “peripheral” members who contribute in more narrow or specialized ways to collective work. A variety of studies have demonstrated that the characteristics of core members can have an outsize impact on teamwork and effectiveness (Humphrey et al., 2009; Mathieu et al., 2014). For example, Pearsall and Ellis (2006) found that the assertiveness of core members had a significant influence on the performance of student teams completing a decision-making simulation, whereas the assertiveness of non-core members did not. Similarly, in a study of Major League baseball teams, Humphrey et al. (2009) found that the career experience of pitchers and catchers—the two roles involved in almost every defensive play in a game—was more strongly related to overall team performance than the career experience of other players on the field. Conversely, just as some attributes of core members can have a large positive impact on team outcomes, other attributes can have an outsized negative impact. For instance, in a study of National Basketball Association teams, those with more-narcissistic point guards (i.e., the position often most central to a team’s offense) exhibited significantly worse coordination, as well as less improvement in coordination over time with increased team familiarity, compared to teams whose point guards were less narcissistic (Grijalva et al., 2020).

Connecting these findings demonstrating the influence of core member characteristics with the evidence of the benefits of member cognitive versatility, we propose that the cognitive style versatility of core team members will be particularly influential for team effectiveness in dynamic settings, especially settings requiring adjustment to changes in role sets.

### Adapting to fluid participation

The research on cognitive versatility, discussed above, demonstrates a variety of potential benefits to including such individuals on teams, and we have further argued that cognitive versatility could be particularly helpful for core team members. We extend this line of reasoning further to suggest that a core member’s cognitive versatility may be especially beneficial in settings involving fluid participation that affects the role sets within teams.

**Figure 1:**
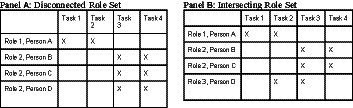
Illustrations of teams disconnected and intersecting role sets. The teams illustrated in Panels **(A,B)** have the same team size and both can accomplish the full set of Tasks 1–4. However, in Panel **(A)**, Roles 1 and 2 have no overlap in the tasks they can do. In Panel **(B)**, Roles 1 and 3 have overlap in their ability to do Task 2, while Roles 2 and 3 also have overlap in their ability to do Task 3.

As mentioned in the introduction, it has become increasingly common for organizations to use roles as an organizing mechanism (Okhuysen and Bechky, 2009), whereby roles provide individuals with clear expectations for their own work and an understanding of their interdependencies with other roles. Clearly defined role sets can allow for coordination despite fluid participation in that specific people may come and go so long as each requisite role is filled (Bechky, 2006; Valentine and Edmondson, 2015). However, just as changes in membership can cause difficulties in teamwork (e.g., see Lewis et al., 2007), changes in the configuration of member skills, leading to changes in the role set, can also disrupt teamwork. Even when the requisite number of members with the necessary skills are present, a change in the configuration of skills across members can lead to the reconfiguration of the role set, which contributes an additional source of disruption. While any change could create difficulty, we contend that when individuals are frequently reassigned to new temporary teams, teams formed with intersecting role sets, with overlap in the capabilities and expectations of different roles, can be particularly disruptive relative to more disconnected role sets (see Figure 1). Intersecting role sets include built-in coordination and adaptation mechanisms by creating more opportunities for backup behaviors, whereas disconnected role sets lack these connections and make backup behavior by team members less likely (Porter, 2005; Bechky and Okhuysen, 2011).

A disconnected role set may be particularly likely to affect the work of the core member of the team, who must navigate the bulk of coordination demands and yet no longer has the same structural flexibility available for task delegation. We theorize, though, that the negative impact of a disconnected role set just described can be mitigated by the presence of a cognitively versatile member in the team’s core position. As discussed above, cognitively versatile core members can enhance team effectiveness as a result of their abilities to think flexibly. They may be able to identify solutions such as re-prioritizing tasks or re-deploying physical resources—solutions other than those related to task-delegation—that could facilitate adaptation to dynamic and demanding work settings. Thus, in dealing with a disconnected role set that is disruptive in part because it limits structural flexibility, cognitively versatile members occupying core roles can offer another mechanism (cognitive flexibility rather than structural flexibility) for flexibly responding to the dynamic environment, thus mitigating the negative impact of missing a role.

Taken together, in the study presented below we will test the following hypotheses:

*Hypothesis 1*: The core member’s (attending physician’s) cognitive style versatility is positively associated with team effectiveness.

*Hypothesis 2*: A disconnected role set (i.e., lacking a nurse practitioner) is negatively associated with team effectiveness.

*Hypothesis 3*: The negative relationship between a disconnected role set (i.e., lacking a nurse practitioner) and team effectiveness is mitigated by the team’s core member’s (attending physician’s) cognitive style versatility.

We test these hypotheses in a field study conducted in an emergency department of a community hospital, a dynamic setting with fluid participation. Below, we introduce our research setting and study design before presenting our analyses and discussing related implications for future research.

## Methods

### Research setting and sample

**Figure 2:**
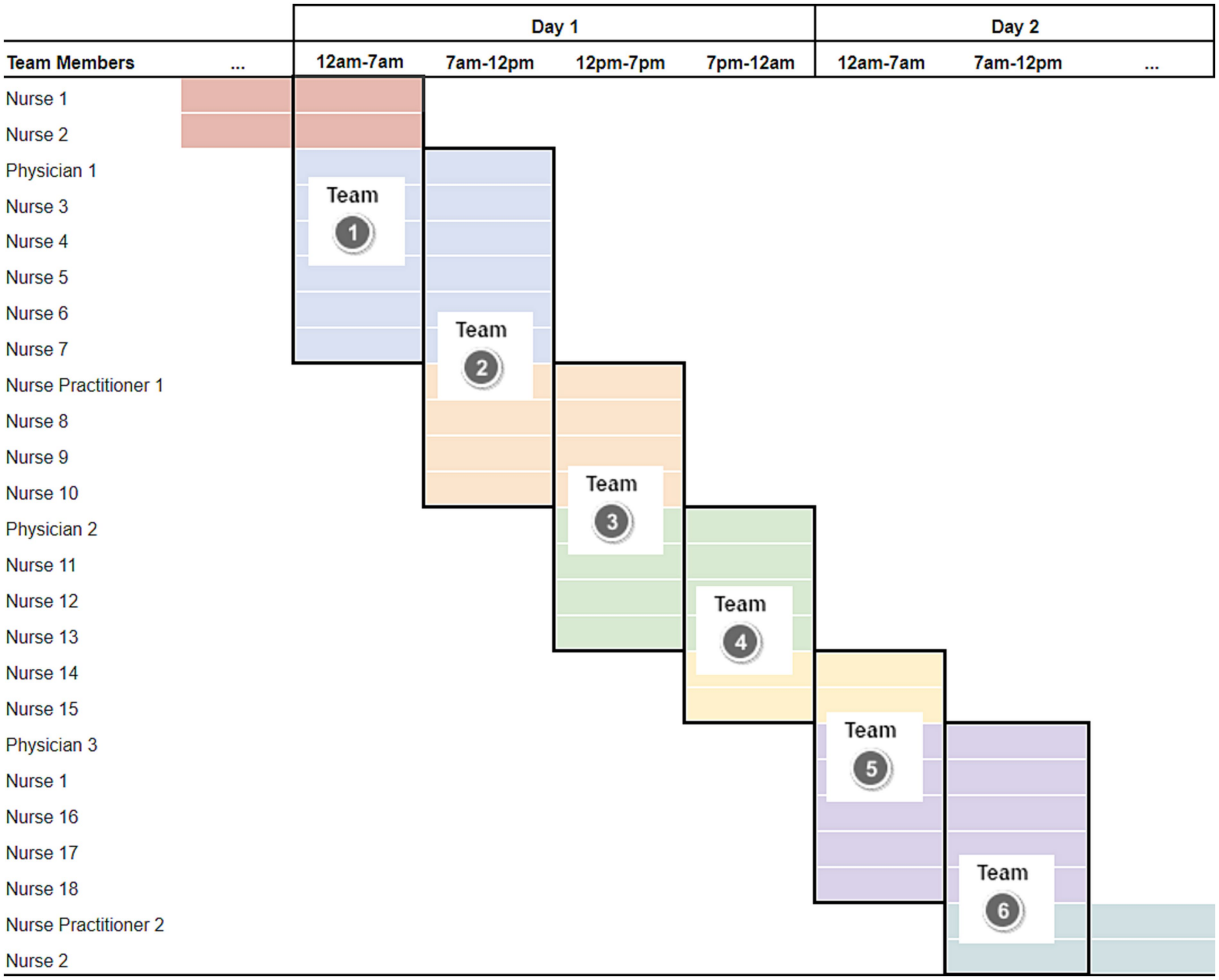
Illustration of approach for defining teams for analysis based on shift schedules. Black outlines within each column indicate members of a team. Different color shading indicates different 12-h shifts. Individuals can repeat in the data. For instance, Nurse 1 appears in teams 1, 5, and 6.

Data for this study come from a 12-bed emergency department (ED) in a community hospital on the West coast of the United States. Data were collected over a period of 5 months during the first half of 2011. Patients visiting this ED were treated by teams consisting of one attending physician, several nurses (average per team = 7), and, for some shifts, one nurse practitioner. As is typical of scheduling in many EDs, physicians and nurses are scheduled in overlapping shifts to facilitate continuity of care for patients by mixing providers who are familiar with current cases with those just starting their shift. For the purposes of defining the teams we use as our unit of analysis for this study, we carve each 24 h period into 4 blocks, demarcated by the shift changes involving some providers, and consider the set of providers working together during a given block of time as a “team” (see Figure 2; each column indicates one team) and account for the overlap/lack of independence of different teams resulting from the carryover of members in our analyses (as we address further in the “Results” section). Defined in this manner, our dataset includes seven physicians working on a total of 342 teams. Individuals working a particular shift 1 day did not necessarily work the same shift subsequently, such that the individuals composing each team varied and the familiarity of team members varied from team to team.

#### Team composition

Of note, in this setting, as in many other hospitals, staffing is planned based on anticipated demand, and shifts that were expected to be busier were more likely to include NPs. In this setting, that meant that the nurse practitioner (NP) position was typically staffed on shifts between noon and 7 pm. Additionally, just as NPs were assigned when demand was expected to be higher, so, too, were nurses, such that the number of nurses tended to be greater when the NP position was staffed. This lack of random assignment raises a concern for the current study. Namely, any observed effect of the NP on our outcomes of interest could be driven not by the *role* of the NP but by having more labor available (from the NP themselves or more nurses). That said, our data allow for a couple of steps to address this empirically, from control variables in our main analyses to a robustness test that employs a coarsened exact matching approach to balance the sample with regard to the number of nurses (as well as the team’s total number of patients). We elaborate on these steps where relevant in our reporting of the results.

### Measures

The data for the study come from a combination of hospital scheduling records, patient health records, as well as surveys completed by attending physicians.^1^

#### Disconnected role set (lacking a nurse practitioner)

While all hospital shifts included an attending physician and several nurses, about 50% of the shifts also included a nurse practitioner NP. The nurse practitioners (NPs) were staffed at times of expected higher patient volumes to ease the burden on the team’s attending physician; similarly, more nurses tended to be staffed during these shifts of higher anticipated patient volume. Regardless of the role configuration, the team members had to work interdependently in providing patient care.

While the three roles were distinct, there was overlap in what the roles could do when an NP was present, as the NPs were trained initially as nurses and could do many tasks that a physician otherwise would do. In contrast, when teams lacked an NP, the roles of attending physician and nurse had little overlap in the tasks they could and were expected to do. We thus considered this role configuration without an NP to reflect a *disconnected role set*. These teams with a disconnected role set had less structural flexibility in the way that tasks could be delegated. Using hospital scheduling records, we created a *disconnected role set* variable for each team which was coded as 1 when the team lacked an NP and 0 when the team included an NP.

#### Core member cognitive versatility

For each of these teams, we considered the attending physician to be the “core” team member because they had the ultimate decision-making authority in this setting and were involved in every case their team handled. Each attending physician in the sample completed the 45-item Object-Spatial Imagery and Verbal Questionnaire (OSIVQ; Blazhenkova and Kozhevnikov, 2009) assessing their strength on the object visualization, spatial visualization, and verbalization cognitive styles. We computed the standard deviation of each attending physician’s scores across the three cognitive style dimensions measured by the OSIVQ to capture the extent to which the attending physician on each team exhibited varied versus similar levels of facility across cognitive styles. We transformed this measure for each individual by multiplying it by −1 so that higher scores indicate greater *core member cognitive versatility*, and we report these scores in Table 1.^2^

**Table 1:** Descriptive statistics and correlations.

				Correlations								
		*M*	SD	1	2	3	4	5	6	7	8	9
1	Core member cognitive style versatility (reverse of the standard deviation)	−3.73	1.50									
2	Disconnected role set (1 = no NP)	0.50	0.50	0.00								
3	Avg. ALOS	−0.04	0.38	**−0.16**	−0.10							
4	Patients handed off	7.78	4.27	**−0.30**	**−0.42**	**0.56**						
*Controls*												
5	Number of nurses	7.06	2.08	0.01	**−0.85**	0.10	**0.35**					
6	Patients carried over from prior team	7.11	4.26	**0.23**	**−0.50**	**0.18**	**0.16**	**0.43**				
7	Admissions	14.16	7.24	**−0.26**	**−0.67**	**0.24**	**0.79**	**0.54**	**0.17**			
8	Average typicality of admissions	111.57	21.77	−0.05	−0.08	0.00	−0.02	0.06	0.00	0.00		
9	Team’s 28-day familiarity	8.14	2.53	**0.47**	**0.46**	−0.10	**−0.48**	**−0.41**	0.01	**−0.55**	0.01	
10	Core member cognitive style strength (mean)	47.61	1.50	−0.50^a^	0.00	**0.17**	**0.29**	0.08	**−0.15**	**0.22**	**−0.13**	**−0.29**
11	Core member social perceptiveness	25.65	5.65	−0.43^a^	0.00	0.01	**0.12**	−0.01	**−0.17**	**0.16**	0.08	**−0.27**
12	Core member conscientiousness	5.78	1.33	0.62^a^	0.00	**−0.12**	**−0.23**	−0.05	**0.20**	**−0.20**	**0.11**	**0.29**
13	Core member experience (Years)	19.46	12.5	0.32^a^	0.00	0.01	**−0.16**	0.03	**0.25**	**−0.24**	−0.04	**0.44**

Bolded coefficients are significant at *p* < 0.05. *N* = 342 unless otherwise indicated; ^a^*n* = 7 for intercorrelations of core member attribute control variables with core member cognitive versatility. Intercorrelations among core member attribute control variables (9–12) are not shown due to the small sample at the individual level (vs the team-level analysis).

#### Team effectiveness

In an ED, a common indicator of performance is the total time elapsed between when a patient arrives and when they are discharged, as this measure is strongly correlated with patient outcomes (Casalino et al., 2012; Valentine and Edmondson, 2015). Since a patient’s primary diagnosis plays a large role in influencing how long a patient stays in the ED (as more complicated problems would require a longer stay), this time must be interpreted in the context of the patient’s diagnosis to create a measure of *adjusted length of stay (ALOS)*, where the time for a given patient is normalized based on the average for patients with the same diagnosis. Based on patient health records, we calculated each team’s *average adjusted length of stay* based on all the patients the team admitted.^3^ As calculated, lower scores signal higher team effectiveness, as they indicate more efficient treatment compared to patients with similar diagnoses. As an additional performance indicator, we also calculated the *number of patients handed off* to the next team, as these are cases that the team initiated but did not resolve before the team was reconstituted due to a shift change. While the overlapping work schedules of doctors and nurses meant that some team members remained involved with the case, the introduction of new providers in a healthcare setting always increased the risk of error either as a result of omitted details in the hand-off and/or gaps in coordination within the newly constituted team, even if some of the original providers remained involved in the case. Therefore, best practices in healthcare often include avoiding handoffs across shifts as much as possible, thus handing off more cases to the next team can be a signal of less effective teamwork.

#### Control variables

We controlled for a number of variables in our analyses that could be alternative explanations for the relationships observed. First, we controlled for a series of attributes of the core member (attending physician) that are often correlated with performance in extant research.^4^ These included *conscientiousness*, measured here using the TIPI (Gosling et al., 2003); *social perceptiveness*, measured with the “Reading the Mind in the Eyes” test (Baron-Cohen et al., 1985); and *experience*, assessed as the individual’s self-reported years spent working as an attending physician. In addition, as is recommended practice when analyzing the effect of a measure of variation, we also controlled for the combined mean of a physician’s scores on the three cognitive styles, termed *core member cognitive strength*.

In addition, we controlled for a series of team-level variables demonstrated to influence team effectiveness in prior work. We controlled for the team’s *familiarity* (Reagans et al., 2005), calculated as the average number of teams on which each dyad worked together in the past 28 days, as team familiarity is often associated with team performance.^5^ We also controlled for factors affecting the team’s workload including the *number of patients carried over from the prior team* (patients that were admitted by a prior team but not yet discharged out of the ED, either home or to the inpatient unit) and the *number of admissions* (patients admitted to the ED during the team’s shift together). Along with these we controlled for the total *number of nurses* on the team as well as the presence of other staff supporting the team during their work such as an *ED technician*, *nurse assistant*, and *patient ambassador* (each coded 1 if present, 0 if absent) as having more support staff can reduce team workload. We also controlled for another factor that can affect workload, the *average case typicality of admitted patients* for each team, based on the frequency with which the primary diagnostic categories of the patients treated by a particular team were observed in the dataset during the timeframe of the study. Case typicality provides an important complement to overall workload and ALOS since dealing with more atypical or unfamiliar diagnoses offers a different challenge to a healthcare team than dealing with a large number of cases or with cases that are complex but familiar.

Finally, we included fixed effects for whether the team was working at *night* (7-midnight and midnight-7 am = 1; else = 0), as well as the team’s *weekday* and *month* to account for related variations in the types of cases handled in the ED (e.g., weather-related accidents, flu season, etc.).

## Results

Descriptive statistics and correlations are reported in Table 1. We estimated a series of Mixed Effects (or Random Coefficient) Models that include random effects for the physicians; we did so using R’s lmer function (see Table 2).^6^ In all of the results reported, we test the hypothesized effects on both of the team effectiveness measures described — *number of patients handed off (handoffs)* and average *adjusted length of stay (Avg. ALOS)*, where for each variable, lower scores are better, indicating greater team effectiveness.

**Table 2:** Mixed-effects models predicting team effectiveness.

	Dependent variable							
	# Patients handed off				Avg. Adj. Length of stay			
	(1)	(2)	(3)	(4)	(5)	(6)	(7)	(8)
Core member cognitive versatility	−0.759*** (0.216)	−0.461* (0.235)	0.051 (0.376)	0.313 (0.360)	−0.107*** (0.032)	−0.079* (0.036)	−0.017 (0.039)	0.009 (0.038)
Disconnected role set (1 = no NP)	3.227*** (0.693)	1.161 (0.966)	3.059*** (0.685)	0.936 (0.957)	0.314** (0.104)	0.119 (0.146)	0.277** (0.102)	0.066 (0.144)
Core member cog. versatility * disconnected role set		−0.552** (0.182)		−0.568** (0.182)		−0.052^+^ (0.028)		−0.056* (0.028)
# Admissions	0.525*** (0.034)	0.538*** (0.034)	0.522*** (0.034)	0.536*** (0.034)	0.021*** (0.005)	0.022*** (0.005)	0.020*** (0.005)	0.021*** (0.005)
# Patients received from prior team	0.157*** (0.043)	0.163*** (0.042)	0.153*** (0.042)	0.161*** (0.042)	0.023*** (0.006)	0.024*** (0.006)	0.023*** (0.006)	0.023*** (0.006)
Avg. typicality of cases	0.004 (0.006)	0.004 (0.006)	0.005 (0.006)	0.004 (0.006)	0.001 (0.001)	0.001 (0.001)	0.001 (0.001)	0.001 (0.001)
# RNs	0.102 (0.173)	0.090 (0.170)	0.058 (0.171)	0.047 (0.169)	0.025 (0.026)	0.024 (0.026)	0.017 (0.026)	0.015 (0.025)
ED tech (1 = yes)	0.025 (0.449)	0.146 (0.445)	0.077 (0.449)	0.205 (0.445)	0.036 (0.067)	0.047 (0.067)	0.049 (0.067)	0.062 (0.067)
Nurse assistant (1 = yes)	−0.123 (0.352)	−0.084 (0.348)	−0.097 (0.352)	−0.055 (0.348)	−0.022 (0.053)	−0.018 (0.053)	−0.018 (0.053)	−0.013 (0.052)
Patient ambassador (1 = yes)	0.142 (0.535)	0.156 (0.528)	0.216 (0.533)	0.225 (0.526)	−0.045 (0.080)	−0.044 (0.080)	−0.032 (0.080)	−0.031 (0.079)
Avg. Familiarity over 28 days	−0.278*** (0.081)	−0.199* (0.084)	−0.257** (0.081)	−0.176* (0.084)	−0.010 (0.012)	−0.002 (0.013)	−0.005 (0.012)	0.003 (0.013)
Core member experience	0.102*** (0.024)	0.095*** (0.024)			0.012** (0.004)	0.011** (0.004)		
Core member social perceptiveness	−0.111*** (0.031)	−0.104*** (0.031)			−0.013** (0.005)	−0.012** (0.005)		
Core member Conscientiousness	0.049 (0.318)	0.083 (0.315)			−0.020 (0.048)	−0.017 (0.048)		
Core member cog. style strength	−0.398 (0.387)	−0.330 (0.383)	0.071 (0.332)	0.085 (0.311)	−0.061 (0.058)	−0.055 (0.058)	0.017 (0.036)	0.018 (0.034)
Constant	16.803 (19.245)	13.750 (19.027)	−2.758 (15.306)	−3.210 (14.333)	2.016 (2.884)	1.727 (2.876)	−1.524 (1.669)	−1.566 (1.567)
Fixed effects (weekday, month, night team)	Yes	Yes	Yes	Yes	Yes	Yes	Yes	Yes
Random effects (Physician)	Yes	Yes	Yes	Yes	Yes	Yes	Yes	Yes
Observations	342	342	342	342	342	342	342	342
Log likelihood	−786.28	−782.53	−786.43	−782.43	−188.39	−189.27	−181.96	−182.56
AIC	1,630.58	1,625.06	1,624.85	1,618.86	434.78	438.54	415.92	419.12
BIC	1,741.78	1,740.10	1,724.56	1,722.40	545.99	553.58	515.63	522.66

^+^*p* < 0.1; *p < 0.05; **p < 0.01; ***p < 0.001. This table reports results for the primary analyses, revealing a generally positive association between a core member’s (Physician’s) cognitive versatility and team effectiveness in that it is associated with fewer handoffs and shooter average lengths of stay, a generally negative association between having a disconnected role set in which the team lacks a nurse practitioner and both team effectiveness measures, and an interaction effect whereby the harmful association between the disconnected role set and team effectiveness is less harmful when the core member is more cognitively versatile.

First, we find that, once controlling for other individual differences, above and beyond those other individual differences, the core member’s cognitive versatility is associated with fewer handoffs to the next team (Table 2, Model 1; *B* = −0.76, *p* < 0.001) and a shorter average ALOS for the patients the team admitted (Model 5; *B* = −0.11, *p* = 0.001).^7^ This supports Hypothesis 1.

Second, we find that having a disconnected role set is associated with more handoffs (see Table 2, Model 1; *B* = 3.23, *p* < 0.001) and a longer average ALOS (Model 5; *B* = 0.31, *p* = 0.002) compared to teams operating with an intersecting role set,^8^ supporting Hypothesis 2.^9^

**Figure 3:**
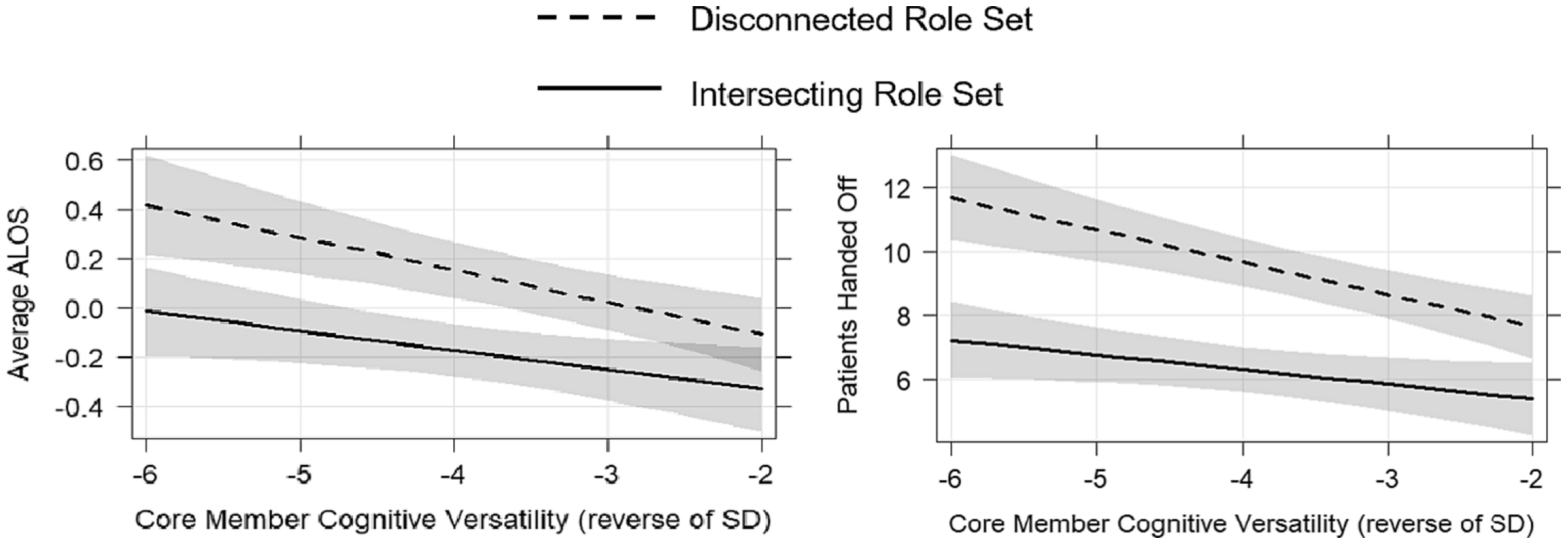
Team effectiveness by core member (Physician) cognitive versatility and role set connectedness (absence of a Nurse Practitioner). The lower scores for Average ALOS (on the left) and the number of patients handed off (on the right) indicate greater team effectiveness. For both outcomes, having a disconnected role set (i.e., missing a Nurse Practitioner) is less harmful if the core member’s (Attending Physician’s) cognitive versatility is greater.

Finally, consistent with Hypothesis 3, the core members’ cognitive versatility moderates the negative association between having a less-connected role set and the number of handoffs (Table 2, Model 2; *B* = −0.55, *p* = 0.002), and this is robust to the exclusion of physician characteristics that we treat as control variables (Model 4, *B* = −0.57, *p* = 0.002). The core member’s cognitive versatility also moderates the negative effect of a disconnected role set on average ALOS, an effect that is significant based on standard thresholds when excluding the physician characteristics that we treat as control variables (Model 8; *B* = −0.06, *p* = 0.041), but did not quite reach significance when including those controls (Table 2, Model 6; *B* = −0.05, *p* = 0.058). In examining the patterns of relationships in a bit more detail (see Figure 3), we observe that the benefit of core member cognitive versatility for both measures of team effectiveness is significantly stronger in teams with a disconnected role set. Moreover, at high levels of cognitive versatility, the benefit of having an intersecting role set is significantly diminished, particularly for average ALOS, such that either having an intersecting role set or a cognitive versatile core team member might afford similar efficiency. To put it in more concrete terms, the findings indicated that when a team has a disconnected role set, a core member with one standard deviation greater cognitive versatility than another team, it would hand off approximately one and a half fewer patients per shift. Given the inefficiency and potential errors introduced when patients are handed off to new healthcare providers, the high financial costs of extending a patient’s stay in the hospital, as well as the value of making beds available for other patients in the ED, reducing the stay for just one patient could be quite consequential.

### Robustness tests

#### Other core member attributes

We hypothesized and found support for predictions based on our theory that the cognitive versatility of a core team member enables that member to think more flexibly and connect with diverse others in order to adapt accordingly. We interpreted our findings showing that core member cognitive versatility moderates the negative relationship impact of a disconnected role set on team efficiency as supporting that argument—that the unique capabilities cognitively versatile core members bring facilitate that adaptation. However, there could always be other reasons why a core member improves team efficiency, and so we examine a few competing explanations as a means of probing our theory about why cognitive versatility is helpful.

Two other individual characteristics that extant research demonstrates are beneficial for teamwork are social perceptiveness and conscientiousness (Riedl et al., 2021; Homan and van Kleef, 2022). Social perceptiveness relates to an individual’s ability to pick up on subtle nonverbal cues and draw inferences about what others are thinking or feeling (Baron-Cohen et al., 1985) which has, like cognitive versatility, been shown to facilitate team coordination, but is orthogonal to cognitive style. Similarly, given all of the details that must be managed in order to treat patients in an ED setting, undoubtedly the conscientiousness of physicians will influence at least some aspects of their performance. But the attention to detail that is often part of individual conscientiousness is not the same as thinking flexibly about those details to adapt to changes that need to be made to get work done. That said, these characteristics could be correlated highly enough that the effects we are attributing to cognitive versatility are in reality the result of these other, correlated characteristics. To examine whether our findings are robust to these potential alternative explanations, we first analyzed whether core members’ social perceptiveness and conscientiousness moderate the relationship between a disconnected role set and average ALOS using two separate models, one for each interaction, wherein the interaction is added to the variables in Table 2, Model 4. We then conducted the same analysis focused on the effects on patient handoffs. In the analysis of the effects of core member social perceptiveness, we observed that the core member’s social perceptiveness did *not* moderate the relationship between a disconnected role set and either average ALOS (*B* = −0.01, *p* = 0.105) or handoffs (*B* = −0.08, *p* = 0.110). In our analysis of core members’ conscientiousness, we observed that it also did *not* moderate the relationship between a disconnected role set and average ALOS (*B* = −0.07, *p* = 0.080); however, conscientiousness did significantly moderate the negative effect of a disconnected role set on handoffs (*B* = −0.65, *p* = 0.009). We interpret these observations to suggest that cognitive versatility captures a unique ability with respect to social perceptiveness, supporting the idea that the ability to pick up on subtle social cues (social perceptiveness) is not enhancing teamwork in quite the same way as the ability to interpret a variety of different ways individuals might convey information and to think flexibly across the different related perspectives (cognitive versatility). Similarly, our analysis of conscientiousness suggests that there may be some unique ways, above and beyond conscientiousness, that cognitive versatility is contributing to more efficient teamwork. That said, individuals’ attention to detail and adherence to requirements (conscientiousness) may coexist with their ability to accurately comprehend and flexibly communicate this information (cognitive versatility).

#### Robustness of varying time frames to analyze effects of familiarity

As another robustness check, we conducted an alternative analysis of the effect of familiarity on team effectiveness using a shorter look-back window; rather than 28 days, we reduced the time frame to 7 days. The use of a shorter look-back window enables us to use a larger sample of teams for analysis (due to having to set aside fewer observations to serve as the look-back window for the initial time period of our analysis) and it’s possible that the increase in statistical power we have in this larger sample would reveal a significant effect we did not observe in our initial analysis. However, our key findings with respect to familiarity remain unchanged (see Table 3).

**Table 3:** Mixed effects models predicting team effectiveness using shortened lookback window for familiarity measure.

	Dependent variable			
	# Patients handed off		Avg. Adj. Length of stay	
	(1)	(2)	(3)	(4)
Core member cognitive versatility	−0.780*** (0.167)	−0.480** (0.186)	−0.089*** (0.026)	−0.064* (0.030)
Disconnected role set (1 = no NP)	3.373*** (0.599)	1.384^+^ (0.827)	0.310** (0.095)	0.145 (0.132)
Core member cog. versatility * disconnected role set		−0.531*** (0.154)		−0.044^+^ (0.025)
# Admissions	0.556*** (0.028)	0.566*** (0.028)	0.020*** (0.005)	0.021*** (0.005)
# Patients received from prior team	0.181*** (0.035)	0.187*** (0.035)	0.023*** (0.006)	0.023*** (0.006)
Avg. typicality of cases	0.002 (0.005)	0.002 (0.005)	0.001 (0.001)	0.001 (0.001)
# RNs	0.094 (0.145)	0.081 (0.143)	0.014 (0.023)	0.012 (0.023)
ED tech (1 = yes)	0.292 (0.363)	0.397 (0.360)	0.026 (0.057)	0.034 (0.057)
Nurse assistant (1 = yes)	0.128 (0.291)	0.137 (0.288)	0.015 (0.046)	0.016 (0.046)
Patient ambassador (1 = yes)	−0.683 (0.444)	−0.622 (0.439)	−0.092 (0.070)	−0.087 (0.070)
Avg. familiarity over 7 Days	−0.857*** (0.169)	−0.675*** (0.175)	−0.041 (0.027)	−0.026 (0.028)
Core member experience	0.090*** (0.018)	0.086*** (0.018)	0.010*** (0.003)	0.010*** (0.003)
Core member social perceptiveness	−0.075** (0.025)	−0.071** (0.025)	−0.010* (0.004)	−0.010* (0.004)
Core member conscientiousness	−0.060 (0.244)	−0.005 (0.242)	0.013 (0.039)	0.018 (0.039)
Core member cog. style strength	−0.519^+^ (0.297)	−0.429 (0.295)	−0.020 (0.047)	−0.012 (0.047)
Constant	22.025 (14.920)	17.981 (14.791)	0.032 (2.360)	−0.304 (2.362)
Fixed effects (weekday, month, night team)	Yes	Yes	Yes	Yes
Random effects (Physician)	Yes	Yes	Yes	Yes
Observations	479	479	479	479
Log likelihood	−1,01.18	−1,096.26	−267.69	−268.88
AIC	2,260.36	2,252.52	593.39	597.76
BIC	2,381.34	2,377.68	714.37	722.91

^+^*p* < 0.1; *p < 0.05; **p < 0.01; ***p < 0.001. This table reports a replication of the primary models reported in Table 2; here the results use a measure of familiarity that is based on a shorter look-back window (7 vs. 28 days). Because fewer observations had to be retained to score the familiarity measure based on this shorter look-back window, these results are based on a larger sample size (479) relative to Table 2 (342).

#### Robustness via coarsened exact matching

Finally, we observed that the absence of an NP was intended to correlate with a lighter workload and, indeed, in our data set the absence of an NP was associated with an overall lower patient load as a result of fewer holdovers from the prior team and fewer admissions. As noted in the description of team composition, the absence of an NP was also associated with having fewer nurses (driven also by wanting fewer staff when there is less work), and greater team familiarity (a byproduct of the generally smaller teams). To address concerns that the effect a disconnected role set, and the moderating effect of cognitive style diversity, might be attributed to factors other than missing the NP, we use the R function MatchIt to implement coarsened exact matching and generate a more balanced data set regarding a patient load variable (the sum of admissions and holdovers) and the number of nurses (which is the main source of variation in team size). Given that the team’s familiarity was largely a function of the number of nurses, matching only on these two variables yielded a balance also on team familiarity. Although the resulting data set retained just 39% of the original data set (*n* = 132), estimations of mixed-effects models using the matched data set yield results consistent with our main analyses. The core member’s cognitive versatility is associated with both fewer handoffs at the next shift change (*B* = −1.16, *p* < 0.001; Table 4, Model 1) and a shorter average adjusted length of stay (*B* = −0.17, *p* < 0.001; Table 4, Model 3). In contrast, having a disconnected role set (lacking a NP) is not significantly associated with our outcomes of interest, but the direction of the effects are consistent with our primary analyses (*B* = 1.65, *p* = 0.103; *B* = 0.11, *p* = 0.424, see Table 4, Models 1 and 3, respectively). Most critically, the interaction effect of the physician’s cognitive versatility and a disconnected role set is significantly associated with both handoffs at the next shift change (*B* = −1.59, *p* < 0.001; Table 4, Model 2) and the average adjusted length of stay (*B* = −0.09, *p* = 0.020; Table 4, Model 4), such that a disconnected role set is less harmful when a team has a more cognitively versatile physician.

**Table 4:** Mixed effects models predicting team effectiveness using matched data.

	Dependent variable			
	# Patients handed off		Avg. Adj. Length of stay	
	(1)	(2)	(3)	(4)
Core member cognitive versatility	−1.156*** (0.342)	−0.308 (0.331)	−0.172*** (0.049)	−0.128^+^ (0.067)
Disconnected role set (1 = no NP)	1.646 (1.009)	−4.306** (1.341)	0.108 (0.135)	−0.248 (0.202)
Core member cog. versatility * disconnected role set		−1.593*** (0.271)		−0.095* (0.041)
# Admissions	0.638*** (0.071)	0.693*** (0.063)	0.016^+^ (0.010)	0.020* (0.009)
# Patients received from prior team	0.021 (0.078)	0.031 (0.068)	0.007 (0.010)	0.008 (0.010)
Avg. typicality of cases	−0.017 (0.014)	−0.008 (0.012)	0.000 (0.002)	0.000 (0.002)
# RNs	−0.509 (0.319)	−0.618* (0.278)	−0.020 (0.043)	−0.025 (0.042)
ED tech (1 = yes)	2.126 (1.541)	2.074 (1.341)	0.170 (0.206)	0.163 (0.201)
Nurse assistant (1 = yes)	−0.662 (0.722)	−0.357 (0.630)	−0.028 (0.096)	−0.015 (0.095)
Patient ambassador (1 = yes)	2.919 (2.245)	1.431 (1.971)	−0.287 (0.300)	−0.357 (0.297)
Avg. familiarity over 28 days	−0.302^+^ (0.160)	−0.093 (0.144)	−0.022 (0.021)	−0.009 (0.022)
Core member experience	0.137*** (0.041)	0.141*** (0.036)	0.018** (0.006)	0.020** (0.007)
Core member social perceptiveness	−0.159** (0.054)	−0.165*** (0.047)	−0.023** (0.008)	−0.024* (0.011)
Core member conscientiousness	0.343 (0.521)	0.282 (0.454)	−0.001 (0.072)	−0.010 (0.085)
Core member cog. style strength	−0.219 (0.592)	−0.321 (0.516)	−0.019 (0.081)	−0.033 (0.088)
Constant	12.963 (29.487)	17.951 (25.687)	0.639 (4.025)	1.358 (4.428)
Fixed effects (weekday, month, night team)	Yes	Yes	Yes	Yes
Random effects (Physician)	Yes	Yes	Yes	Yes
Observations	132	132	132	132
Log likelihood	−315.69	−301.03	−104.41	−104.09
AIC	689.38	662.06	266.83	268.17
BIC	772.98	748.55	350.43	354.66

^+^*p* < 0.1; *p < 0.05; **p < 0.01; ***p < 0.001. This table reports a replication of the primary models reported in Table 2; here the results are based on a matched data set created using coarsened-exact matching to balance the data set on a patient load variable (the sum of admissions and holdovers) and the number of nurses (which is the main source of variation in team size). Thirty-two percent of the original sample was retained in this process (*n* = 132).

In sum, the primary analyses that include control variables, when taken together with the robustness tests reported above, provide results consistent with our theory that the impact of a disconnected role set, which omits the NP role, presents a challenge to team coordination that is not explained by having more or less labor, having more or fewer nurses, specifically, or having a greater/lesser workload.

## Discussion

In our study of hospital Emergency Department (ED) teams, we find that disconnected role sets, measured as the absence of a Nurse Practitioner (NP) such that there is less overlap in the tasks that roles could do, is associated with less team effectiveness, measured in terms of patients’ length of stay in the ED and patient handoffs at the next shift change. However, we also find that the cognitive style versatility of the strategically core team members (i.e., the attending physician) is associated with greater team effectiveness and mitigates the negative relationship between a disconnected role set and team effectiveness. These findings have implications for the Carnegie School, research on fluid teams, and research on intrapersonal diversity.

### Carnegie School

The work of the Carnegie School and the work it inspired has uncovered a variety of organizational structures that can guide attention and coordination to support more effective collective work. This is true, too, in teams, where role structures can support the coming and going of individuals. Yet, with increasingly fluid participation in teams, teams are beginning to resemble the organized anarchies they once were used to control. Indeed, the garbage can model’s element of fluid participation (Cohen et al., 1972) that has largely gone underdeveloped has emerged anew in the study of teams (e.g., Mortensen and Haas, 2018), where team forms are so fluid as to call into question what constitutes a team today (Wageman et al., 2012), but where scholars nonetheless have turned attention to understanding what might support these teams to enable the effective teamwork today’s organizations demand (Mayo, 2022). By integrating across the Carnegie School and teams research, drawing from psychology and organizational behavior research to do so, we move from typical structures (e.g., roles), or even the network properties of role structures (e.g., the connectedness of role sets) to consider another fundamental lever in team design (team composition), uncovering another possible antidote to the chaos of organized anarchy in the form of member cognition.

### Fluid teams

Research on teams has increasingly been grappling with the reality of fluid participation in teams (e.g., Edmondson, 2012; Mortensen and Haas, 2018; Mayo, 2022). While role-based team structures have been shown to offer one mechanism of support to fluid teams (e.g., Bechky, 2006; Valentine and Edmondson, 2015), the current study highlights one condition under which this mechanism falls short: when the fluidity of participation alters the set of roles available such that a team must work with a disconnected role set. This could emerge due to limited role availability as a result of organizational decisions to not make a role available at all times, as in the case of nurse practitioners. In short, we extend from past work suggesting that role-based systems work in part because they offer clear understanding of how to coordinate; here, we highlight how having a role set with more overlap in the tasks the roles can perform might also be critical for adaptation in that it allows for members to back up one another, creating a structural mechanism for some flexibility. Indeed, that seems to be why, in health care, the nurse practitioner role emerged at all (Berg, 2020). In short, such roles afford the team some structural flexibility. Removing such a role, which may be sensible at times from the perspective of staffing costs, requires that members reconsider how they are allocating their attention while having less structural flexibility to handle their collective workload. We both highlight this as an organizational problem and suggest one antidote in the form of core team member’s cognitive style versatility.

Beyond the possibility that an intersecting role set creates structural flexibility useful for adaptation, the presence of certain roles might offer the added benefit of serving as a bridge between other members. For instance, in our empirical setting of emergency departments, the healthcare industry has created a variety of new positions, or roles, over time. This expanding set of roles is typically considered to either allow for more targeted care via positions that are increasingly specialized in their training, or to allow for more effective access to care via positions that require less training, are less expensive, and can ease the demand on more specialized and expensive positions. The NP role we focused on is an example of the latter. While already noted above that the NP is capable of (and allowed to do) many tasks otherwise delegated to a physician, NPs also share a common training with registered nurses in that NPs are first trained as registered nurses. NPs thus could serve as a sort of broker between physicians and nurses, helping to bridge a divide rooted in training and professional status that has long been acknowledged to detract from patient care (Bransby et al., 2023). Future work on fluid participation thus may do well to take a contextualized approach to the problem at hand (Johns, 2006).

Our focus was notably on disconnected role sets that were planned, as could arise with, for instance, scheduled vacation time. However, surprises could yield the same outcome, such as when a swat team loses a member mid-operation (Bechky and Okhuysen, 2011), someone takes sick leave, or a scheduling conflict arises, for instance, due to their participation in multiple ongoing teams, forcing individuals to choose where to allocate attention. Future work could explore these unanticipated shifts in available roles.

### Intrapersonal diversity

Our focus on cognitive versatility is an example of a way in which an individual exhibits intrapersonal diversity, and we contribute to the growing research on intrapersonal diversity, broadly, in multiple ways. First, we theoretically focus here on the potential mechanisms of cognitive versatility in terms of the ability for cognitively versatile members to think flexibly. This theorizing is consistent with research that has documented that other forms of intrapersonal diversity (e.g., having intrapersonal diversity in functional area or cultural experiences) can fuel greater breadth and less rigidity in information processing (Bunderson and Sutcliffe, 2002; Jang, 2017). Critically, extant research suggests that intrapersonal diversity operates by offering individuals multiple lenses through which to view and interpret the world (e.g., Maddux et al., 2021), much like is achieved with cognitive style versatility. Moreover, above we noted that, in our setting, the NPs could facilitate connection across other roles. Here, too, other forms of intrapersonal diversity have been shown to breed greater communication competence and an ability to bridge diverse team members or subgroups and resolve conflict (Mok and Morris, 2010; Marian and Shook, 2012; Jang, 2017; Aggarwal et al., 2019; Mell et al., 2021; Maddux et al., 2021; Lu et al., 2022). Collectively, these prior studies suggest that individuals with intrapersonal diversity as a result of their background and diverse experiences bring a variety of valuable attributes to teams as a result of their own cognitive flexibility, their enhanced communication competence, and their capacity to use these skills to facilitate information sharing and integration. Future work to tease out the mechanisms of intrapersonal diversity in general, and cognitive versatility in particular, could be fruitful.

Second, we integrate a specific focus on cognitive style versatility with a role-based view of team composition. This integration allowed for uncovering that strategically core team member’s greater cognitive versatility may allow for adaptation, and specifically in the face of disconnected role sets. In our specific setting of emergency department teams, where the core member is the attending physician, cognitive versatility could facilitate the flexible thinking noted to be required for, to name few examples, adapting to changing clinical scenarios or deviating from a protocol when necessary, re-prioritizing patients as a team as the panel of patients changes and individual patient needs evolve, and redistributing limited tools (e.g., monitors, mobile computers) throughout the team (Ward et al., 2006). Indeed, our definition of the physician as the core role reflects the fact that they hold decision-making authority, which positions the attending well to make these adjustments if he or she notices the need to do so and can identify possible solutions. While we do not have data that would allow us to identify the underlying mechanism in our setting, our findings are consistent with this theory, and suggest a future path for exploring the importance of intrapersonal diversity among core team members.

Further, we found that the relationship between strategically core members’ cognitive style versatility and team effectiveness held above and beyond other well-studied personality and social attributes such as conscientiousness and social perceptiveness. We also find that the core member’s social perceptiveness was associated with team effectiveness, as we might expect (e.g., see Riedl et al., 2021), but it did not moderate the relationship between a disconnected role set and team effectiveness. While we hesitate to over-interpret this null finding, it bolsters our interpretation of our findings. We theorized that disconnected role sets hinder performance because they limit the requisite adaptation for performing in volatile contexts, such as an ED. As such, if a core role holder’s cognitive versatility moderates the impact of a disconnected role set then it is likely doing so via adaptation; this adaptation is not something we would necessarily expect social perceptiveness to facilitate (Baron-Cohen et al., 2001) and so the lack of a significant interaction is consistent with our logic. It is also possible that the core role holder’s conscientiousness, a personality trait that is a strong predictor of taskwork (Homan and van Kleef, 2022) plays a similar role in fluid teams. Empirical evidence, however, did not support its effects on either team effectiveness measure, but core member conscientiousness did significantly mitigate the adverse effect of a disconnected role set on patient handoffs. It could be that core team members who are more conscientious are also more likely to attend to who is doing what, such that they ensure that no tasks are dropped, something perhaps at greater risk when the role that creates role intersections is missing. Nonetheless, accounting for this interaction did not significantly change the impact that cognitive versatility had on the same outcome, indicating that these characteristics are likely to operate in different ways in how they impact team effectiveness. Future work could explore the interplay of various traits of team members.

## Limitations

There are multiple limitations to this work that we would be remiss not to mention. First, we note that this work is only correlational. While we have attempted to account for alternative explanations for our effects with our control variables and robustness tests, we caution that these results be interpreted as suggestive of the role of intrapersonal diversity given the possibility of endogenous factors that may not be fully accounted for here (particularly the risk of omitted variable bias). Future work to unpack the causal effects of intrapersonal diversity on team adaptation to the chaos of fluid participation is needed. Second, we speculate that cognitive versatility may affect the possible solution set that a core member is able to identify and choose from when facing general problems in a dynamic work environment, as well as the specific challenge of a less-connected role set. However, we do not have data that would allow us to observe this mechanism. Future work to assess this possible mechanism could shed further light on interventions that could support a fluid team’s work irrespective of its core member’s cognitive style versatility. Similarly, we speculate that a disconnected role set can affect the team’s capacity to coordinate, but here, too, we do not have data that would allow us to observe coordination behaviors. Future work to explore the specific impacts of changes in the overall role set could be fruitful given the amount of work that is both role-based and fluid in today’s organizations.

## Conclusion

The Carnegie School laid an impressive foundation for a profound variety of fields of study, let alone topics of study within psychology and organizational behavior. The early identification of attention and fluid participation as key factors that could influence effective organizing rings as true today as it did when first developed, and perhaps even more so in the teams literature given new forms of organizing that stretch members’ attention, in part because of increased fluidity. This study contributes to the Carnegie tradition, then, by connecting research from psychology and organizational behavior to uncover one possible antidote to the organized anarchies that are teams today.

## Data availability statement

The raw data supporting the conclusions of this article will be made available by the authors, without undue reservation.

## Ethics statement

The studies involving humans were approved by Carnegie Mellon University Institutional Review Board. The studies were conducted in accordance with the local legislation and institutional requirements. The participants provided their written informed consent to participate in this study.

## Author contributions

IA, BA, EZ, and AW contributed to the conception or design of the work. IA, BA, and AW contributed to data collection. IA, AM, TM, and EZ contributed to data analysis and interpretation. IA and AM contributed to drafting the article. All authors contributed to critical revision of the article and approved publication of the content.

## Footnotes

^1^Non-physician roles were also surveyed on two occasions; however, there are a substantial number of these non-physicians for whom surveys were not administered.

^2^A substantial literature on cognitive versatility includes considerable debate about how it is best measured (Sadler-Smith, 2009). In recent work examining cognitive versatility in teams, team-level cognitive versatility was indexed based on the number of members whose scores exceeded a specific threshold on more than one cognitive style dimension, where the number of members meeting those criteria served as one indicator of team-level cognitive versatility (Aggarwal et al., 2023). By contrast, here we focus on the impact of the level of cognitive versatility of one specific team member, the core role holder, and theorize benefits related to higher (versus lower) levels of the cognitive versatility exhibited by that individual. Therefore, we use a continuous measure based on the standard deviation across the three dimensions to enable us to accurately capture the level of cognitive versatility of the core member of each team. We also replicated our effects using the coefficient of variation, which some have suggested is particularly well-suited for questions of asymmetrical dispersion (Bedeian and Mossholder, 2000; Harrison and Klein, 2007) – a pattern that is of theoretical interest to us. Results are consistent in direction and significance when using this alternative measure.

^3^Some patient stays in the ED extended across multiple teams; we attribute length of stay for a patient to the team that first saw the patient in the ED, as the initial diagnostic and care plan this team originates has a large influence on how efficiently a case is handled overall.

^4^We also explored two additional models. First, we estimated an OLS model using fixed effects for physicians to control for all characteristics of the physicians that remain stable during our observation window. In doing so, we were limited to testing hypotheses 2 and 3. Results are consistent with the model we present and reported in the Appendix. Because we sought to differentiate cognitive versatility and its moderating effect from other specific individual differences, we have opted to report as our primary models those that include specific physician features (e.g., social perceptiveness, etc.) rather than include only fixed effects for the physicians. Second, we also estimated mixed-effects models with cluster-robust standard errors, clustered by the physician with a method suggested for small sample sizes (Imbens and Kolesár, 2016; Pustejovsky and Tipton, 2018). Our results are unchanged for the effects related to H1 and H2, although they do change for H3 (*B* = −0.55, *p* = 0.36, and *B* = −0.05, *p* = 0.42, for the interactions predicting handoffs and ALOS, respectively). That said, we take the fact that the mixed-effects models do not address the clustering of both physicians and other roles, the significant findings for H1-H3 in the multiple membership models that do account for that clustering when estimating standard errors (Zhang et al., 2016), and the significant findings from the fixed effects models regarding H2-H3, to collectively offer reason to believe that the evidence for not just H1 and H2, but also H3 is compelling.

^5^We chose a 28-day lookback window following recent work demonstrating familiarity effects based on a lookback window of this length (Kim et al., 2023). The length of the lookback window has implications for the amount of data available for analysis, as longer windows require us to reserve more data to provide one period to “look back” on at the beginning of the study time period. We present robustness checks using lookback windows of different lengths to ensure our findings are robust to this decision.

^6^The data notably violate an assumption of this hierarchical model in that both physicians *and* individuals in other roles can appear in the data as members of multiple teams (i.e., individuals never worked on more than one team at the same time, but they did work on multiple teams during our data collection window). To account for the subsequent lack of independence between teams, we also estimated multiple membership models (Browne et al., 2001) using the R package R2MLwiN. We report the results in the Appendix. Because the key findings are consistent in direction and significance, we report the simpler models here for ease of interpretation.

^7^We note that these effects are only significant in Models 1 and 5, where we control for other core-member individual differences (as opposed to Models 3 and 7).

^8^We note that these effects are significant whether controlling for all core-member individual differences (Models 1 and 5) or not (Models 3 and 7).

^9^Of note, these effects emerge when controlling for the number of nurses on the team, which helps to address any concern that the effect of the NP is not due to the NP role, *per se*, but that this staffing often co-occurs with more nurse staffing. Additionally, the presence of the NP implies an extra person available on the team, and thus the impact of having the NP could be simply having more labor (not from nurses, but the NP themselves), rather than something about the team’s role composition. Thus, we conducted additional analyses in which we explored the role of team size measured as the sum of the number of nurses, the physician, and the nurse practitioner, if present. As with our primary analyses, we find that the effects we predicted are observed above and beyond this team size measure, which itself was not associated with average ALOS or handoffs. We further note that cognitive versatility does not moderate the effect of either the number of nurses or the team size on average ALOS or handoffs. Finally, we further address this concern below in a robustness test using a coarsened exact matching approach.
