# Supplementary material for: Cognitive versatility and adaptation to fluid participation in hospital emergency department teams
Source: Front Psychol. 2024 Feb 27;15:1144638. doi: 10.3389/fpsyg.2024.1144638 (PMC10927813; doi:10.3389/fpsyg.2024.1144638)
Supplement: Supplementary file 1 [file Table_1.docx]

# Appendix

As noted in footnote 6 in the main text, the data here violate a key assumption in our mixed-effects models: namely, the units of analysis (teams) are not independent because members of one team can and typically do appear in the data as a member of other teams given the continuous reconstitution of teams in this setting. While we account for the nesting of teams into physicians in our mixed-effects models, this fails to account for the additional nesting into other roles. Multiple membership models (Browne et al., 2001) that can account for this were estimated using the R package R2MLwiN. Results reveal that the study’s key findings regarding both patient handoffs and length of stay are consistent in direction and significance (see Table A1). These models suggest that, after accounting for specific core-member attributes, the variance explained by team members is minimal.

## Table A1: Multiple Membership Models


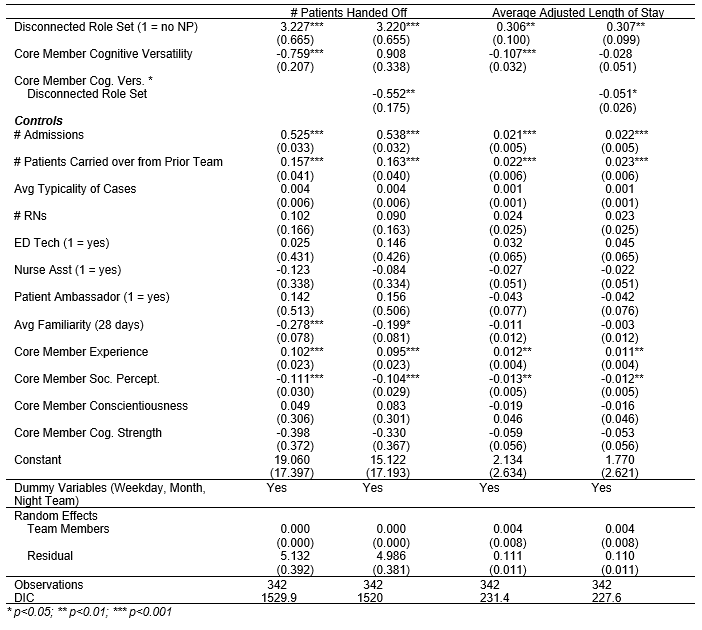


As noted in Footnote 4, we further test hypotheses 2 and 3 using an OLS model with fixed effects for the physicians. This allows us to control for characteristics of the physicians that remain stable during our observation window, including those we measured as well as unobserved characteristics such as intelligence or physical fitness. We estimated simple linear models using R’s lm function and including dummy variables for the physician. Results lend further support for our hypotheses. First, consistent with hypothesis 2, a disconnected role set, measured in terms of lacking a nurse practitioner (NP), leads to more handoffs and longer average adjusted lengths of stay for patients (average ALOS; Table A2, Models 1 and 3, respectively). Second, consistent with hypothesis 3, the harmful effect of a disconnected role set (i.e., of lacking a NP) on the number of patients handed off to the next team and average ALOS, is mitigated by having a more cognitively versatile physician (Table A2, Models 2 and 4, respectively), though we note that the interaction effect on average ALOS did not reach the standard threshold of statistical significance.

## Table A2: OLS Models with Fixed Effects for Physicians

**
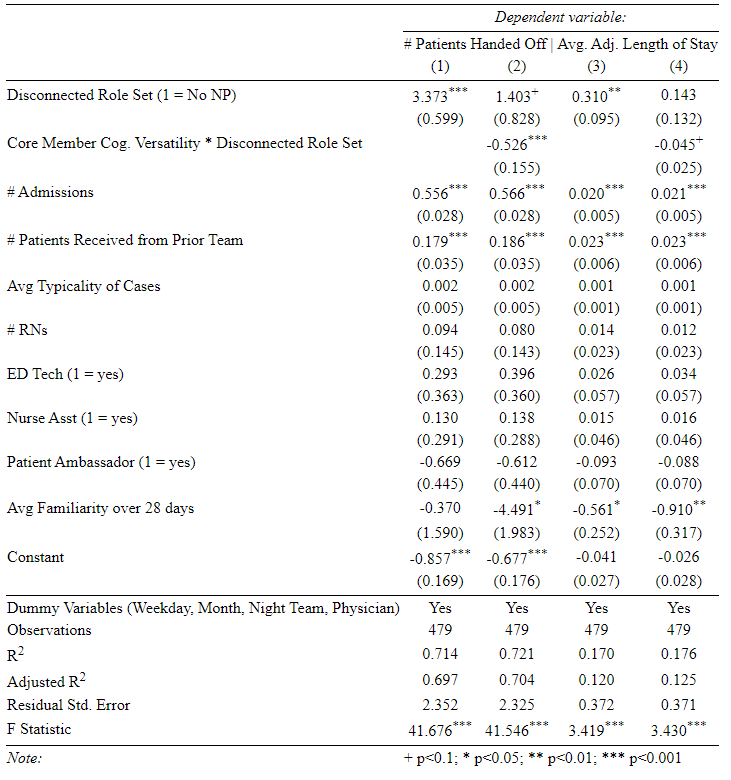
**
